# Updated Reference Limits for Liver Blood Tests With Validation Against Long‐Term Liver‐Related Outcomes

**DOI:** 10.1111/liv.70440

**Published:** 2025-11-19

**Authors:** Fredrik Åberg, Antti Jula, Veikko Salomaa, Annamari Lundqvist, Satu Männistö, Markus Perola, Ville Männistö

**Affiliations:** ^1^ Transplantation and Liver Surgery Helsinki University Hospital and University of Helsinki Helsinki Finland; ^2^ Finnish Institute for Health and Welfare Helsinki Finland; ^3^ Department of Medicine University of Eastern Finland Kuopio Finland; ^4^ Department of Medicine Kuopio University Hospital Kuopio Finland

**Keywords:** ALT, cirrhosis, liver enzyme, prediction, transaminase

## Abstract

**Background and Aims:**

Liver blood tests are widely used, but their reference limits may not reliably predict long‐term liver‐related risks. Previous studies to establish reference limits often lacked rigorous exclusion of individuals with undiagnosed liver disease or associated risk factors. This study aims to establish more precise reference limits in a rigorously defined healthy middle‐aged population and validate their clinical relevance against major adverse liver outcomes (MALOs).

**Methods:**

We analysed 5412 participants from the Finnish population‐based Health 2000 Survey, systematically excluding individuals with baseline or future liver disease during the next 10 years, advanced fibrosis, liver‐related risk factors, hepatotoxic medication use, or systemic conditions to define a truly healthy reference population. Alanine aminotransferase (ALT), aspartate aminotransferase (AST), gamma‐glutamyl transferase (GGT), alkaline phosphatase (ALP), and bilirubin were measured using standard methods. External validation was conducted in three independent FINRISK cohorts (*n* = 20 423) against incident MALOs.

**Results:**

The updated reference limits (97.5th percentile) in the healthiest subpopulation for men and women, respectively, were: ALT 57 and 35 U/L, AST 49 and 33 U/L, GGT 48 and 75 U/L, ALP 108 and 93 U/L, and total bilirubin 28 and 25 μmol/L. A 10‐year MALO risk of > 5% was observed for AST > 2× and GGT > 3× the upper reference limit, whereas no ALT elevation exceeded this risk level. At AST > 2× the upper reference limit, the MALO risk surpassed or equalled competing mortality risk.

**Conclusions:**

Updated reference limits for liver blood tests were established in a well‐characterised population and validated against liver outcomes, with AST and GGT proving to be stronger predictors than ALT.

**Summary**

- The appropriate “normal” limits for liver blood tests are still debated.
- We studied a truly healthy group from the Finnish population—excluding anyone with hidden liver disease or who later developed it—to set updated, more accurate limits.
- These new limits can help doctors decide more reliably when further investigations into liver health are needed.

**Abbreviations**
ALP: alkaline phosphatase
ALT: alanine aminotransferase
AST: aspartate aminotransferase
GGT: gamma‐glutamyl transferase
HILMO: Care Register for Health Care
ICD: International Classification of Diseases
IFCC: International Federation of Clinical Chemistry
MALO: major adverse liver outcome
NORIP: Nordic Reference Interval Project

## 1 Introduction

Liver blood tests, including alanine aminotransferase (ALT), aspartate aminotransferase (AST), gamma‐glutamyl transferase (GGT), alkaline phosphatase (ALP), and bilirubin, are commonly used laboratory tests in clinical practice [1]. These biomarkers, especially transaminases, play a key role in routine healthcare to detect liver disease and injury, monitor the effects and side effects of various drug therapies, and assess general health during regular check‐ups. Despite their popularity, the establishment of accurate and clinically meaningful reference limits remains challenging [2, 3, 4, 5]. Furthermore, validating these thresholds against long‐term liver‐related clinical outcomes is crucial to ensure clinical relevance, yet this has been largely overlooked in previous studies [4].

Current reference limits are often derived from populations that did not rigorously exclude individuals with relevant risk factors or subclinical and undiagnosed liver disease using modern methods. Many studies fail to incorporate markers of liver fibrosis or longitudinal follow‐up data to validate thresholds against clinical outcomes. There is concern that this methodological gap may have inflated the reference limits in earlier studies, potentially reducing their sensitivity [2, 4]. Concerns have also been raised that current reference ranges for some analytes, such as ALT, may be too high, which could lead to underdiagnosis of both early and advanced liver disease [6, 7]. Although most research has focused on ALT [2, 8, 9, 10], less attention has been given to determining appropriate reference limits for other common liver biomarkers, such as AST, GGT, ALP, and bilirubin.

Although current European guidelines [11, 12] recommend fibrosis testing in individuals with persistently elevated liver enzymes, they do not define explicit thresholds for these enzymes. This gap further underscores the need to refine population‐based reference limits that are clinically meaningful and evidence‐based.

To address these limitations, our study aims to establish more precise reference limits for ALT, AST, GGT, ALP, and bilirubin in a large, population‐based cohort. By systematically excluding individuals with known liver disease, steatosis or advanced fibrosis, relevant risk factors, medication use, and incident liver‐related events during the 10 years after measurements, we defined a truly “healthy” reference population. Furthermore, we validated the updated reference limits in independent population cohorts, linking them to long‐term liver‐related clinical outcomes to ensure their clinical relevance.

## 2 Material and Methods

### 2.1 Participants

For the purpose of establishing reference limits for the biomarkers, we used data from the Health 2000 Survey, a multidisciplinary epidemiological study performed in Finland in 2000–2001, which was coordinated by the Finnish Institute for Health and Welfare (previously known as the National Public Health Institute of Finland) [13]. Because of the regional two‐stage stratified cluster sampling procedure, the sample is considered representative of the entire Finnish population. Baseline data were gathered via structured interviews and questionnaires, clinical measurements, blood tests, and clinical examinations by a physician [14]. The Epidemiology Ethics Committee of the Helsinki and Uusimaa Hospital Region approved the Health 2000 Survey protocol, and all participants provided signed informed consent. In 2015, the Health 2000 Survey sample was transferred to THL Biobank after approval from the Coordinating Ethics Committee of the Helsinki and Uusimaa Hospital District and the Ministry of Social Affairs and Health.

**FIGURE 1:**
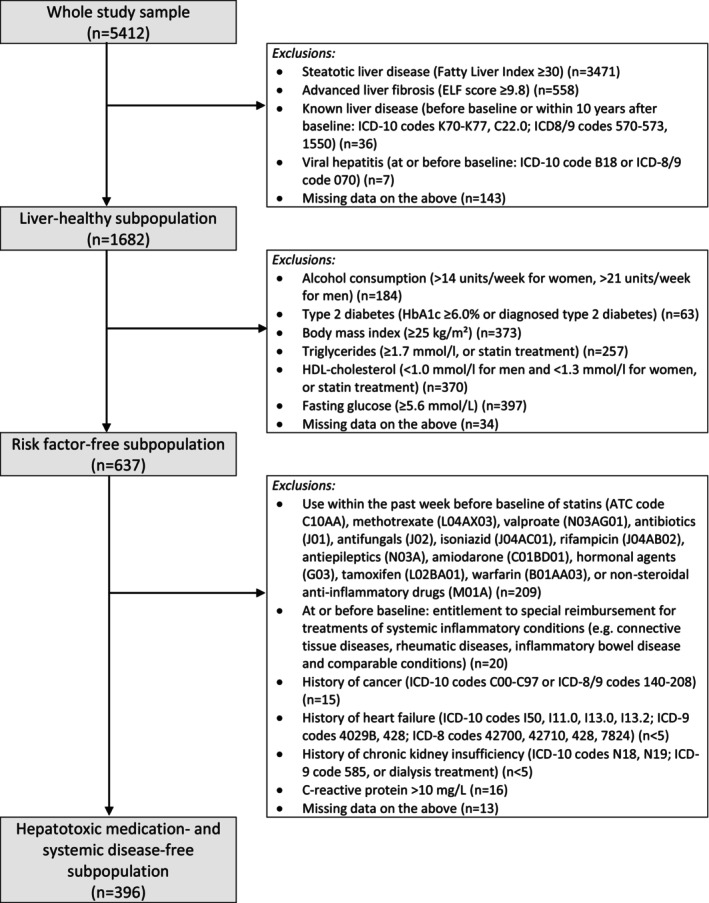
Flow chart showing the exclusion criteria for the various subpopulations of the Health 2000 study sample. Any subject may have several exclusion criteria simultaneously.

Of the original sample of 8028 adults aged 30 years or more, the participation rate was 84% and 6078 (76%) subjects were invited and participated in the full health examination and had blood samples available for biomarker analyses. After excluding 347 individuals with lipemic or hemolytic blood samples, 295 individuals aged > 80 years, and 24 pregnant women, the overall study sample comprised 5412 individuals. From this overall sample, we defined several subpopulations in this study on the basis of the increasing rigor of screening for liver disease and associated risk factors (Figure 1).

### 2.2 Laboratory Analyses

Analyses were performed from serum samples collected after a minimum of 4 h fasting. The sampling, storage and processing of these samples have been described in detail in the Health 2000 methodology report [14]. Serum samples were immediately frozen to −20°C on site, normally within 45–60 min but no later than 90 min from sampling. The samples in the boxes were packed in dry ice and transferred from the field storage points to their final storage location (−70°C), no later than 1–2 weeks after sampling. ALT, AST, ALP, and total bilirubin measurements were analysed in the year 2021 from the serum samples collected at baseline. The laboratory measurements were carried out at the Biochemistry Laboratory, Dept. of Government Services, Finnish Institute for Health and Welfare, Helsinki. The testing laboratory (T077) is accredited by the Finnish Accreditation Service (FINAS) and it fulfills the requirements of the standard SFS‐EN ISO/IEC 17025:2005. Serum ALT was measured by the Activated Alanine Transferase assay and AST by the Activated Aspartate Transferase assay according to the recommendations of the assay manufacturer (Abbott Laboratories, Abbott Park, IL, USA), using a clinical chemistry analyser, Architect c8000 (Abbott Laboratories, Abbott Park, IL, USA). The reagents used are based on optimised formulations recommended by the International Federation of Clinical Chemistry (IFCC). GGT was analysed as part of the Health 2000 survey at the Analytical Biochemistry Laboratory of the National Public Health Institute from serum samples by a Kone Optima 909 analyser (Thermo Electron, Vantaa, Finland) using the kinetic method (Labsystems, Espoo, Finland) according to European recommendations ECCLS.

### 2.3 Registry Linkage

Survey data were linked to the Care Register for Health Care (HILMO) for hospitalisation data, the Finnish Cancer Registry for malignancies, Statistics Finland for causes of death, and the Finnish Drug Reimbursement Register for entitlement to special drug reimbursement. The law mandates data collection in these registries; coverage and general quality of the data are consistent and complete [15, 16]. Linkage was performed using a unique personal identity code assigned to all Finnish residents. Follow‐up for deaths and hospitalisations was conducted until December 2019, and for malignancies until December 2021.

### 2.4 Subpopulations

The reference ranges for liver analytes were defined in the overall study population and its subpopulations with increasing rigour of screening for liver disease and associated risk factors (Figure 1). First, a liver‐healthy subpopulation was formed by excluding individuals with steatotic liver disease (Fatty Liver Index > 30 [17]), advanced liver fibrosis (Enhanced Liver Fibrosis value ≥ 9.8 [18, 19]), a registry record of any type of liver disease before baseline or within 10 years after baseline, or chronic viral hepatitis at or before baseline. A risk factor‐free subpopulation was then formed from the liver‐healthy subpopulation by additionally excluding individuals with hazardous alcohol use, diabetes, overweight, elevated triglycerides, low HDL‐cholesterol, or increased fasting glucose (Figure 1). Finally, we formed a hepatotoxic medication‐ and systemic disease‐free subpopulation by excluding from the risk factor‐free subpopulation also those reporting to have used potentially liver‐toxic medications within the past week and those with systemic conditions that could potentially affect liver analytes. We also excluded individuals from each subpopulation with missing data on the exclusion criteria. Participants with missing data on exclusion criteria were more often men, slightly older, and socioeconomically more disadvantaged compared to those with complete data (Table S1).

### 2.5 External Validation Dataset

For the purpose of validating the reference intervals of ALT, AST and GGT against incident major adverse liver outcomes (MALOs), we used data from three population‐based FINRISK studies (2002, 2007 and 2012). These cross‐sectional health‐examination surveys conducted by the Finnish Institute for Health and Welfare (previously National Public Health Institute) provide data on representative samples of adults (25–74 years) from 4 to 6 regions in Finland. The samples were randomly drawn from the Finnish National Population Register and were stratified by region, gender, and 10‐year age groups [20]. After excluding individuals with any type of liver disease at or before baseline (ICD‐10: K70‐K77, C22.0), the validation dataset comprised 20 423 individuals with 104 incident MALO events during follow‐up. ALP and total bilirubin were unavailable in this dataset.

MALOs were defined by a registry diagnosis of liver cirrhosis, oesophageal varices (bleeding or non‐bleeding), hepatic ascites, hepatorenal syndrome, portal hypertension, hepatic encephalopathy, chronic liver failure, and hepatocellular carcinoma (Table S2). The ICD codes used here to define MALOs are based on international expert consensus [21].

### 2.6 Statistical Analyses

Reference limits for each liver blood test are reported as the 2.5th and 97.5th percentile on the basis of the Health 2000 Survey overall study population and subpopulations. The final reference limits selected were those obtained in the most stringent subpopulation (hepatotoxic medication‐ and systemic disease‐free subpopulation). Because of the right‐skewed distribution of liver blood tests, we also estimated reference limits after applying log‐transformation separately for men and women to better approximate normality. Reference limits were then defined by calculating 95% confidence intervals on the log scale and back‐transforming the results to the original scale.

In the FINRISK validation dataset, we categorised individuals as those with tests below the lower reference limit, within the reference limits, 1–1.5 times above the upper reference limit, 1.5–2 times, 2–3 times, and > 3 times above the upper reference limit. We then performed univariable Cox regression analyses to analyse the rates of MALOs in these groups. We repeated the Cox regression analysis, adjusting for age, sex, body mass index, weekly alcohol consumption, and diabetes. The proportional hazards assumption was assessed using scaled Schoenfeld residuals, and no violations were detected. We also estimated the 10‐year cumulative probability of MALOs and the competing risk of death without MALOs using the cumulative incidence function. Statistical significance was defined as a two‐tailed P‐value < 0.05. Data were analysed using the R software version 4.3.1. ChatGPT was used for language editing purposes.

## 3 Results

### 3.1 Reference Limits in the Health 2000 Sample

The overall study sample comprised 5412 individuals, the liver‐healthy subpopulation 1682 individuals, the risk factor‐free subpopulation 637 and the hepatotoxic‐ and systemic disease‐free subpopulation 396 individuals (Table 1). Mean age in the overall sample was 51 years, whereas mean age in the hepatotoxic‐ and systemic disease‐free subpopulation was 43 years (Table 1). The corresponding proportion of women in these two populations was 53% and 64%, respectively.

**TABLE 1:** Baseline demographics in the Health 2000 population and subpopulations, as well as in the external validation dataset (FINRISK 2002, 2007 and 2012). Results are presented as mean (SD) or *n* (%).

	Health 2000 data				Validation data (FINRISK)
	Overall population	Liver‐healthy population	Risk factor‐free population	Hepatotoxic medication‐ and systemic disease‐free population	
Number	5412	1682	637	396	20 423
Age	51.0 (13.2)	46.4 (11.7)	44.0 (10.8)	42.6 (10.1)	49.7 (13.7)
Men	2532 (46.8)	566 (33.7)	174 (27.3)	144 (36.4)	9555 (46.8)
Body mass index (kg/m^2^)	27.0 (4.6)	23.1 (2.4)	22.0 (1.8)	22.0 (1.8)	27.0 (4.8)
Diabetes	505 (9.3)	63 (3.7)	0	0	1693 (8.3)
Alcohol use (g/week)	78.3 (146.0)	66.1 (115.3)	37.7 (42.5)	39.5 (44.8)	80.2 (136.4)
HDL‐cholesterol	1.33 (0.37)	1.42 (0.36)	1.38 (0.36)	1.41 (0.37)	1.48 (0.39)
Triglycerides	1.60 (1.02)	1.20 (0.54)	1.28 (0.57)	1.24 (0.53)	1.42 (0.97)
Fasting glucose	5.54 (1.21)	5.29 (0.81)	5.02 (0.29)	5.03 (0.28)	—
Fatty Liver Index	46.6 (27.0)	17.0 (7.5)	14.2 (7.3)	14.3 (7.8)	43.1 (30.9)
Enhanced Liver Fibrosis test	8.8 (0.8)	8.5 (0.6)	8.3 (0.6)	8.3 (0.6)	—
Alanine aminotransferase (U/L)	25.6 (17.9)	20.0 (11.0)	18.1 (10.6)	18.5 (10.7)	27.1 (19.6)
Aspartate aminotransferase (U/L)	27.9 (12.4)	25.4 (8.6)	24.5 (8.7)	24.8 (9.5)	28.3 (19.3)
Gamma‐glutamyltransaminase (U/L)	35.7 (44.2)	22.9 (14.6)	24.8 (16.1)	24.1 (14.9)	34.1 (49.6)
Alkaline phosphatase (U/L)	71.4 (22.2)	65.1 (19.6)	61.3 (17.2)	61.8 (16.9)	—
Total bilirubin (μmol/L)	10.0 (5.8)	9.7 (5.4)	9.9 (5.6)	10.6 (6.2)	—

**FIGURE 2:**
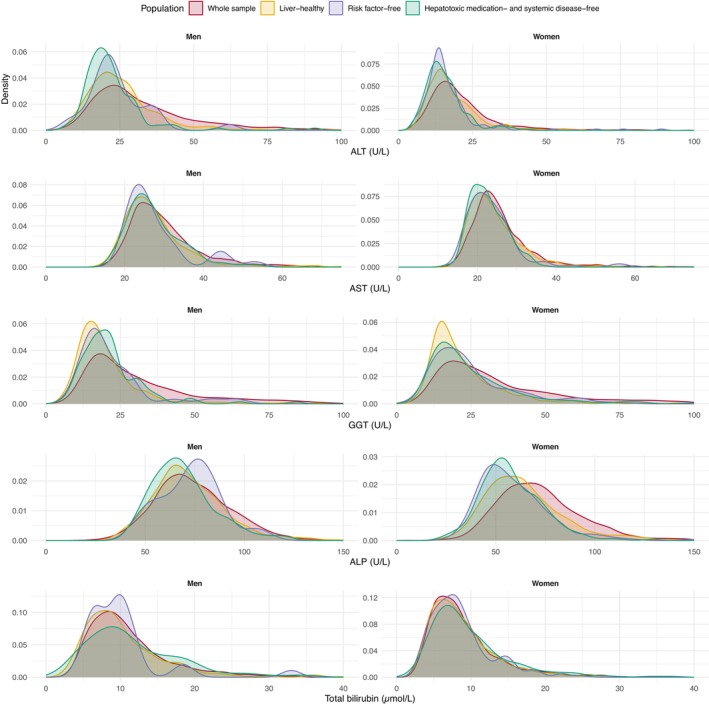
Sex‐stratified distributions of liver blood tests in the various subpopulations of the Health 2000 study.

Median values and reference values (2.5th and 97.5th percentiles) of the liver blood tests by sex and in the various subpopulations are shown in Table 2. Furthermore, sex‐stratified median and reference values according to age groups in the hepatotoxic‐ and systemic disease‐free subpopulation are shown in Table S3. The sex‐stratified distributions of liver blood tests in the various subpopulations are visualised in Figure 2. As expected, applying increasingly stringent exclusion criteria to subpopulations resulted in the removal of more cases with high liver blood test values, whereas median values were only modestly affected.

**TABLE 2:** Sex‐specific median values and 2.5th and 97.5th percentiles (reference limits) for the various liver blood tests in the Health 2000 overall study population and its subpopulations.

Analyte and population	Men				Women			
	*N*	Median	2.5th percentile	97.5th percentile	*N*	Median	2.5th percentile	97.5th percentile
Alanine aminotransferase (U/L)								
Overall population	2421	27	11	84	2707	17	7	50
Liver‐healthy population	533	22	10	57	1034	15	7	39
Risk factor‐free population	166	21	10	58	423	14	6	36
Hepatotoxic medication‐ and systemic disease‐free population	137	21	12	57^a^	226	14	7	35^a^
Log‐transformation^b^			10	47			7	31
Aspartate aminotransferase (U/L)								
Overall population	2532	27	18	61	2880	24	16	48
Liver‐healthy population	566	26	18	51	1116	22	16	40
Risk factor‐free population	174	26	18	50	463	22	16	36
Hepatotoxic medication‐ and systemic disease‐free population	144	26	18	49	252	22	16	33
Log‐transformation^b^			15	48			15	33
Gamma‐glutamyltransaminase (U/L)								
Overall population	2478	23	10	134	2814	24	10	135
Liver‐healthy population	566	18	8	50	1116	20	9	63
Risk factor‐free population	174	19	10	49	463	21	10	71
Hepatotoxic medication‐ and systemic disease‐free population	144	20	10	48	252	20	10	75
Log‐transformation^b^			9	44			8	62
Alkaline phosphatase (U/L)								
Overall population	2532	71	42	120	2880	65	36	119
Liver‐healthy population	566	69	43	115	1116	59	34	109
Risk factor‐free population	174	68	45	108	463	55	33	98
Hepatotoxic medication‐ and systemic disease‐free population	144	67	44	108	252	55	31	93
Log‐transformation^b^			43	105			33	95
Total bilirubin (μmol/L)								
Overall population	2532	10	4	27	2880	8	4	22
Liver‐healthy population	566	9	4	27	1116	8	4	22
Risk factor‐free population	174	10	4	29	463	8	4	23
Hepatotoxic medication‐ and systemic disease‐free population	144	10	4	28	252	8	4	25
Log‐transformation^b^			4	30			3	23

^a^ 95th percentiles for ALT are 41 U/L for men and 27 U/L for women.

^b^ Reference limits were estimated in the hepatotoxic medication‐ and systemic disease‐free subpopulation after applying log‐transformation separately for men and women to better approximate normality. Reference limits were then defined by calculating 95% confidence intervals on the log scale and back‐transforming the results to the original scale.

### 3.2 External Validation for Incident MALOs

In the validation dataset combining three population‐based FINRISK studies (2002, 2007, and 2012), the mean age was 50 years, 53% women, a mean body mass index of 27 kg/m^2^, and 8% had diabetes. Mean ALT, AST, and GGT were 27 U/L, 28 U/L, and 34 U/L, respectively.

By univariable and multivariable Cox regression analysis, the rates for MALOs increased significantly along with increasing ALT, AST, and GGT categories when compared to the subset within reference limits (Table 3). For example, in the subset with ALT > 3 times above the upper reference limit, the unadjusted hazard ratio (HR) for MALO was 10. For AST, the unadjusted HR in the highest category (> 3× upper reference limit) was 42, and for GGT, it was 53, showing the strongest association with MALO. A dose–response relationship was evident across all these categories, with increasing HRs observed even at lower elevations. The trend was similar in multivariable analyses (Table 3).

**TABLE 3:** Hazard ratios for major liver‐related outcome events as well as 10‐year cumulative risks for both liver events and the competing risk of death without liver events in the external validation dataset (FINRISK 2002–2012) according to the magnitude of deviation from the sex‐specific reference limits in three liver blood tests.

	Individuals	Liver events	Unadjusted hazards ratio (95% CI)	Adjusted hazards ratio (95% CI)^a^	10‐year cumulative incidence of liver events (95% CI)	10‐year cumulative incidence of competing‐risk events (95% CI)
Alanine aminotransferase (U/L)						
Below the lower reference limit	273	0	n.c.	n.c.	0.0 (0.0–0.0)	13.6 (9.3–18.0)
Within reference limits	18 049	68	1.00	1.00	0.3 (0.2–0.4)	5.9 (5.5–6.2)
1–1.5 × URL	1257	20	4.41 (2.68–7.26)	3.91 (2.27–6.71)	1.4 (0.7–2.1)	5.0 (3.7–6.3)
1.5–2 × URL	280	< 5	4.05 (1.48–11.10)	3.25 (1.15–9.17)	1.6 (0.0–3.3)	7.1 (3.9–10.3)
2–3 × URL	167	7	12.54 (5.76–27.31)	10.88 (4.60–25.70)	3.2 (0.1–6.4)	7.4 (3.1–11.7)
> 3 × URL	53	< 5	10.44 (2.56–42.59)	14.11 (3.43–58.05)	3.8 (0.0–8.9)	3.8 (0.0–8.9)
Aspartate aminotransferase (U/L)						
Below the lower reference limit	273	0	n.c.	n.c.	0.0 (0.0–0.0)	4.3 (1.8–6.8)
Within reference limits	17 173	49	1.00	1.00	0.2 (0.1–0.3)	5.6 (5.2–6.0)
1–1.5 × URL	1150	21	7.03 (4.22–11.73)	6.33 (3.68–10.89)	1.2 (0.5–1.9)	7.9 (6.2–9.7)
1.5–2 × URL	198	9	18.84 (9.25–38.38)	11.68 (4.92–27.72)	4.1 (1.1–7.2)	9.6 (5.0–14.1)
2–3 × URL	70	8	48.97 (23.18–103.45)	35.90 (15.13–85.18)	12.6 (3.6–21.6)	11.7 (3.3–20.2)
> 3 × URL	54	5	42.17 (16.79–105.92)	49.97 (19.68–126.86)	10.8 (1.4–20.2)	11.1 (2.7–19.5)
Gamma‐glutamyltransaminase (U/L)						
Below the lower reference limit	527	0	n.c.	n.c.	0.0 (0.0–0.0)	1.4 (0.4–2.5)
Within reference limits	17 253	39	1.00	1.00	0.2 (0.0–0.2)	5.3 (5.0–5.6)
1–1.5 × URL	1363	15	5.08 (2.80–9.22)	3.88 (2.04–7.37)	0.7 (0.2–1.1)	9.7 (8.0–11.3)
1.5–2 × URL	554	5	4.34 (1.71–11.00)	2.56 (0.89–7.40)	0.8 (0.1–1.6)	9.2 (7.0–11.7)
2–3 × URL	415	15	18.78 (10.35–34.01)	12.68 (6.52–24.65)	3.1 (1.0–5.0)	13.1 (10.0–16.6)
> 3 × URL	309	30	52.72 (32.73–84.93)	27.57 (15.29–49.72)	8.8 (5.0–12.1)	20.6 (20.1–25.5)

Abbreviations: n.c., not calculable; URL, upper reference limit.

^a^ Adjusted for age, sex, body mass index, weekly alcohol consumption, and diabetes.

The 10‐year cumulative incidence analyses showed that although the relative risks for MALOs increased with higher liver enzyme levels, the absolute risk increases were small (Table 3). Even in the subset with ALT > 3 times above the upper reference limit, the 10‐year risk remained below an arbitrary 5%. In contrast, a > 5% 10‐year risk was observed for AST at levels exceeding 2× the upper reference limit and for GGT at > 3× the upper reference limit. Notably, with AST > 2× the upper reference limit, the 10‐year absolute risks of MALOs equalled or even exceeded those for competing‐risk events (death without MALO), highlighting AST as a particularly strong marker of liver‐related outcomes. This effect was not observed for ALT or GGT, where competing risks remained at least as high as, or higher than, the risk of developing a MALO.

Interestingly, although no MALOs occurred in individuals with liver blood tests below the lower reference limit, those with ALT below this threshold had an increased 10‐year risk of death without MALOs. This pattern was not observed for AST or GGT. ALP and bilirubin were not available in the FINRISK cohorts.

## 4 Discussion

We defined reference limits for multiple standard liver blood tests in a well‐characterised population‐based sample after rigorous exclusion of individuals with liver disease or associated risk factors. We then confirmed the clinical relevance of the reference limits for ALT, AST, and GGT through external validation against incident MALOs in a combined dataset of three general population cohorts, spanning different time periods and including participants without baseline clinical liver disease, with no additional selection criteria.

Strengths of our study include the randomly selected and well‐characterised general population sample with extensive health data and universal liver blood test measurements. At‐risk advanced fibrosis was excluded using the Enhanced Liver Fibrosis (ELF) test, which has a negative predictive value of 98% in low‐prevalence settings [18]. Steatotic liver disease was excluded using the Fatty Liver Index, which has a negative predictive value of 98%–99% [22]. Furthermore, registry linkage enabled the exclusion of individuals who developed liver‐related outcomes within 10 years after baseline, and systematic screening ensured the removal of participants with relevant liver‐related risk factors, systemic conditions or medication usage that may induce liver damage. Although the baseline examinations were conducted in 2000–2001, all liver blood laboratory analyses, except for GGT, were performed in 2021 according to current IFCC laboratory standards. However, despite being conducted over 20 years ago, the GGT analyses used the kinetic method, which remains the standard approach today.

The upper reference limits for ALT found in our study (97.5th percentile) were very similar to those reported in the IFCC study from 2010 [3], 13 units lower for men and 10 units lower for women than in the Nordic Reference Interval Project 2000 (NORIP) study [23], slightly higher than those reported by Valenti et al. [2], and higher than those reported by Prati et al. [10], Lee et al. [9], and Tan et al. [8] (Table 4).

**TABLE 4:** Comparison of upper reference limits of various liver blood tests in the present study and in previous studies. Reference limits estimated in our study using the log‐transformation method are shown in parentheses.

Analyte and study	Men	Women	Percentile used for upper reference limit	Skewness correction
Alanine aminotransferase (U/L)				
Prati et al. 2002 [10]	30	19	95	No
Lee et al. 2010 [9]	35	26	97.5	No
Tan et al. 2023 [8]	36	28	95	Partial (meta‐analysis; bootstrap for pooled percentiles)
Valenti et al. 2021 [2]	42	30	95	No
*Present study*	41	27	95	No
	57 (47)	35 (31)	97.5	Provided in parenthesis
Ceriotti et al. 2010 [3]	59	41	97.5	No
Rustad et al. 2004 [23]	70	45	97.5	Log‐transform for outlier removal
Aspartate aminotransferase (U/L)				
Ceriotti et al. 2010 [3]	35	33	97.5	No
Rustad et al. 2004 [23]	45	35	97.5	Log‐transform for outlier removal
*Present study*	49 (48)	33 (33)	97.5	Provided in parenthesis
Gamma‐glutamyltransaminase (U/L)				
Ceriotti et al. 2010 [3]	68	40	97.5	No, but excluded NORIP outliers
Rustad et al. 2004 [23]	Age 18–39: 80 Age 40+: 115	Age 18–39: 45 Age 40+: 75	97.5	Log‐transform for outlier removal
*Present study*	Overall: 48 (44) Age 18–39: 53 Age 40+: 36–41	Overall: 75 (62) Age 18–39: 79 Age 40+: 60–89	97.5	Provided in parenthesis
Alkaline phosphatase (U/L)				
Rustad et al. 2004 [23]	105	105	97.5	Log‐transform for outlier removal
*Present study*	108 (105)	93 (95)	97.5	Provided in parenthesis
Total bilirubin (μmol/L)				
Rustad et al. 2004 [23]	25	25	97.5	Dixon's rule for outlier removal
*Present study*	28 (30)	25 (23)	97.5	Provided in parenthesis

There are important differences between these studies and populations. The NORIP study recruited subjectively healthy reference individuals from the local populations surrounding the participating laboratories across the Nordic countries [23]. The IFCC study selected reference individuals based on an anamnestic interview conducted by a physician using an “ad hoc” questionnaire [3]. Neither study specifically screened for advanced liver fibrosis or steatotic liver disease using modern diagnostic methods, nor did they include data on incident liver‐related outcomes following the measurements. The study by Prati et al. [10] used an unoptimised ALT assay lacking pyridoxal phosphate and set the upper reference limit at the 95th instead of the 97.5th percentile, while also excluding all ALT values > 40 U/L in males and > 30 U/L in females a priori, leading to lower reference limits. Lee et al. [9] reported similarly low upper reference limits among 1105 Korean potential living‐liver donors with biopsy‐confirmed healthy livers, a notably young cohort with a mean age of 29 years (SD 9.0). Tan et al. [8] determined upper reference limits for ALT through a meta‐analysis of 47 studies, encompassing 423 355 presumably healthy individuals. However, variability in ALT assays, inconsistency in threshold selection (95th vs. 97.5th), predominantly Asian populations, and differences in methodologies and exclusion criteria across the included studies contributed to significant heterogeneity and uncertainty in the derived reference limits. Valenti et al. [2] analysed 21 296 apparently healthy blood donors without liver‐related risk factors using the standardised IFCC methodology. They used the 95th percentile, leading to slightly lower upper reference limits. When using the 95th percentile instead of the 97.5th percentile in our study, the upper reference limits for ALT closely aligned with those reported by Valenti et al. Therefore, methodological choices—particularly in addressing the right‐skewed distribution of ALT—substantially influence the derived upper reference limits.

Fewer studies have analysed reference limits for AST, GGT, ALP, and total bilirubin. Our upper reference limit for AST matched those in the NORIP and IFCC studies for women, but was slightly higher for men, though still within the reported bias range in the NORIP study [3, 23]. The upper reference limit for ALP was slightly lower in women in our study, whereas ALP in men and total bilirubin values were otherwise consistent with those in the NORIP study.

Surprisingly, the upper reference limits for GGT in our study were higher for women than for men, differing from the IFCC and NORIP studies [3, 23]. Although our limits for men were lower than in these studies, those for women were higher. This finding persisted even after skewness correction, applying the 95th percentile, using stricter alcohol exclusion thresholds (e.g., < 70 g/week and < 30 g/week), and excluding women with high waist‐hip ratios (results not shown). GGT is strongly correlated with alcohol use, adiposity, and several other lifestyle and metabolic factors [24], all of which were controlled for in our study. It could be speculated that the strict exclusion criteria disproportionately lowered GGT values among men. Alternatively, residual confounding or true sex‐specific differences under low‐risk conditions may have contributed. The IFCC study also reported considerable regional variability in GGT upper reference limits, with particularly high values in a subset of the Nordic population [3], suggesting possible genetic or analytical influences.

Although liver blood tests above our reference limits were statistically significantly associated with MALOs, the 10‐year absolute risk differences compared to those within reference limits were minimal. This supports the use of slightly higher cutoffs rather than the lower limits reported in some previous studies.

AST > 2 times the upper reference limit and GGT > 3 times the upper reference limit were associated with a 10‐year MALO risk exceeding an arbitrary 5%. In contrast, no ALT level was associated with the 10‐year risk above 5%, though reliance on single measurements and the low number of individuals with ALT > 3 times the upper reference limit weakens the strength of conclusions. Only for AST > 2 times the upper reference limit was the MALO risk equal to or higher than the competing risk. Interestingly, ALT below the lower reference limit was associated with increased non‐MALO mortality, a finding previously reported and possibly attributable to frailty, muscle wasting and malnutrition [25, 26]. No similar association was observed for AST or GGT in our study.

Study limitations include reliance on single liver blood test measurements, which may not capture dynamic changes over time. Furthermore, as the dataset originates from ethnically homogeneous Finnish cohorts, external validity may be limited, and differences in genetic, environmental, or lifestyle factors could influence analyte distributions in other populations. However, although GGT is known to vary regionally, the IFCC study [3] found no meaningful regional variation for other liver blood tests. In Finland, the prevalence of hepatitis C is similar to the EU median (0.6% vs. 0.5%) [27], whereas the prevalence of chronic hepatitis B is lower (0.2% vs. 0.5%) [28, 29].

Although the Health 2000 and FINRISK cohorts are geographically representative of the Finnish adult population, non‐participants typically have lower education, lower occupational class, and poorer health than participants [20, 30, 31]. In addition, Health 2000 participants with missing data on exclusion criteria were more often men, slightly older, and socioeconomically more disadvantaged compared to those with complete data. Although such healthy participant bias may affect the generalisability of absolute risk estimates, we expect minimal impact on the reference limits established using the Health 2000 data, as we applied rigorous exclusion criteria to define a strictly healthy reference population. In the FINRISK‐based validation, potential underestimation of absolute liver‐related risks may occur, but relative risk estimates were adjusted in multivariable models, mitigating confounding.

Validation of ALP and bilirubin was not possible because of a lack of data in the FINRISK cohorts, which limits the strength of the proposed reference limits for these markers. Furthermore, because of the lack of platelet count data, we could not compare the prognostic ability of liver blood tests to FIB‐4. Although FIB‐4 has shown better prognostic performance for MALO than liver blood tests in unselected populations, its overall accuracy remains modest [32], and its use is not recommended in individuals without risk factors for liver disease or elevated liver enzymes. Appropriate reference limits of liver enzymes are also essential for the purpose of targeted FIB‐4 testing.

No widely accepted consensus exists on the upper limit of normal for defining an abnormal liver blood test in clinical decision‐making, and few studies have linked specific test levels to absolute risk for future liver outcomes. Despite strict exclusion criteria to define a healthy population without liver stress factors, our study found higher upper reference limits than some recent reports. Although concerns exist that currently used limits may be too high and could miss at‐risk patients, our findings support the somewhat higher upper reference limits proposed by, for instance, the IFCC study [3] and Valenti et al. [2].

Liver blood tests remain valuable for monitoring drug treatments, but their role in detecting advanced chronic liver disease in the community is diminishing. Standard liver blood tests alone lack discriminatory power for advanced liver disease [33]. For example, 72%–88% of individuals with advanced liver fibrosis in primary care and 91% of those with asymptomatic compensated cirrhosis had normal ALT levels [7].

Although 10%–20% of the population has mildly elevated liver blood tests [33], absolute MALO risks remain low. Inappropriately lowering the upper reference limit would increase false positives, leading to unnecessary testing, referrals, treatments, and patient anxiety. Accurate identification of clinically significant chronic liver disease requires novel diagnostic triggers beyond routine liver blood tests [34, 35].

In conclusion, on the basis of a well‐characterised and representative general population sample, we propose updated reference limits for several standard liver blood tests, ensuring clinically meaningful thresholds linked to long‐term liver‐related outcomes. Notably, AST and GGT exhibited stronger associations with major adverse liver outcomes than ALT, emphasising their relevance in clinical decision‐making in routine practice.

## Ethics Statement

The Epidemiology Ethics Committee of the Helsinki and Uusimaa Hospital Region approved the Health 2000 Survey protocol.

## Consent

All participants provided signed informed consent.

## Conflicts of Interest

The authors declare no conflicts of interest.

## Supporting information


**Table S1:** Comparison of participants in the Health 2000 Survey with complete data or missing data on any of the exclusion criteria used to construct the study subsamples.
**Table S2:**. The ICD codes used to define major adverse liver outcomes.
**Table S3:**. Sex‐specific median values and 2.5th and 97.5th percentiles (reference limits) for the various liver blood tests in the Health 2000 hepatoxic medication‐ and systemic disease‐free population according to age groups.

## Data Availability

The survey data used in this study are available from the THL Biobank on the basis of a research application, as explained on the website of the THL Biobank (https://thl.fi/en/research‐and‐development/thl‐biobank/for‐researchers).
