# Supplementary material for: Updated Reference Limits for Liver Blood Tests With Validation Against Long‐Term Liver‐Related Outcomes
Source: Liver Int. 2025 Nov 19;45(12):e70440. doi: 10.1111/liv.70440 (PMC12628042; doi:10.1111/liv.70440)
Supplement: Supplementary file 1 — Table S1: Comparison of participants in the Health 2000 Survey with complete data or missing data on any of the exclusion criteria used to construct the study subsamples. Table S2:. The ICD codes used to define major adverse liver outcomes. Table S3:. Sex‐specific median values and 2.5th and 97.5th percentiles (reference limits) for the various liver blood tests in the Health 2000 hepatoxic medication‐ and systemic disease‐free population according to age groups. [file LIV-45-0-s001.docx]

Supplementary Appendix

**Updated reference limits for liver blood tests with validation**

**against long-term liver-related outcomes**

Fredrik Åberg, Antti Jula, Veikko Salomaa, Annamari Lundqvist,

Satu Männistö, Markus Perola, Ville Männistö

**Supplementary table 1.** Comparison of participants in the Health 2000 Survey with complete data or missing data on any of the exclusion criteria used to construct the study subsamples.

| **Variable** | **No missingness**  (n = 4065) | **Missing data on at least one exclusion criterion**  (n = 1347) | **P** |
| --- | --- | --- | --- |
| **Age**, mean (SD) | 49.90 (12.81) | 54.39 (13.69) | <0.001 |
| **Women** (%) | 2275 (56.0%) | 605 (44.9%) | <0.001 |
| **Education level, n (%**) |  |  | <0.001 |
| Low | 1381 (34.0%) | 630 (47.4%) |  |
| Average | 1383 (34.0%) | 418 (31.4%) |  |
| High | 1299 (32.0%) | 282 (21.2%) |  |
| **Employment status, n** (%) |  |  | <0.001 |
| Part- or full-time employed | 2590 (63.7%) | 644 (48.4%) |  |
| Other | 438 (10.8%) | 137 (10.3%) |  |
| Retired | 1037 (25.5%) | 550 (41.3%) |  |
| **Marital status, n** (%) |  |  | <0.001 |
| Married/partnership | 2977 (73.2%) | 902 (67.8%) |  |
| Single | 453 (11.1%) | 159 (11.9%) |  |
| Widow/separated | 635 (15.6%) | 270 (20.3%) |  |

**Supplementary table 2.** The ICD codes used to define major adverse liver outcomes.

| **Diagnosis** | **ICD-10** | **ICD-9** | **ICD-8** |
| --- | --- | --- | --- |
| Esophageal varices, bleeding | I85.0, I98.3 | 456.0, 456.20 | 456.0 |
| Esophageal varices, non-bleeding | I85.9, I98.2 | 456.1, 456.21 | 456.0 |
| Ascites | R18 | 789.5 | 785.3 |
| Hepatorenal syndrome | K76.7 | 572.4 |  |
| Liver failure, chronic | K72.1 | 572.8 | 573 |
| Liver cirrhosis | K74.6 | 571.5 | 571.9 |
| Liver cirrhosis due to alcohol | K70.3 | 571.2 | 571.0 |
| Liver encephalopathy |  | 572.2 | 573.02 |
| Liver failure not otherwise defined | K72.9 |  |  |
| Portal hypertension | K76.6 | 572.3 | 571.9 |
| Hepatocellular carcinoma | C22.0 | 155.0 | 155.01 |
| Liver transplantation | Z94.4 | V42 |  |

**Supplementary table 3.** Sex-specific median values and 2.5^th^ and 97.5^th^ percentiles (reference limits) for the various liver blood tests in the Health 2000 hepatoxic medication- and systemic disease-free population according to age groups.

|  | Men | | | | | Women | | | |
| --- | --- | --- | --- | --- | --- | --- | --- | --- | --- |
| Analyte and population | **N** | **Median** | | **2.5th percentile** | **97.5th percentile** | **N** | **Median** | **2.5th percentile** | **97.5th percentile** |
| Alanine aminotransferase (U/L) | | | | | | | | | |
| Age < 40 years | 67 | 22 | | 13 | 85 | 89 | 14 | 6 | 24 |
| Age 40-59 years | 55 | 20 | | 13 | 30 | 114 | 14 | 7 | 34 |
| Age ≥ 60 years | 14 | 16 | | 8 | 35 | 20 | 18 | 9 | 47 |
| Aspartate aminotransferase (U/L) | | | | | | | | | |
| Age < 40 years | 70 | | 26 | 18 | 66 | 102 | 21 | 16 | 30 |
| Age 40-59 years | 59 | | 25 | 18 | 41 | 127 | 22 | 16 | 33 |
| Age ≥ 60 years | 14 | | 24 | 19 | 46 | 20 | 26 | 18 | 41 |
| Gamma-glutamyltransaminase (U/L) | | | | | | | | | |
| Age < 40 years | 70 | | 20 | 8 | 53 | 102 | 22 | 11 | 79 |
| Age 40-59 years | 59 | | 18 | 11 | 41 | 127 | 19 | 9 | 60 |
| Age ≥ 60 years | 14 | | 22 | 10 | 36 | 20 | 20 | 10 | 89 |
| Alkaline phosphatase (U/L) | | | | | | | | | |
| Age < 40 years | 70 | | 67 | 46 | 97 | 102 | 53 | 31 | 95 |
| Age 40-59 years | 59 | | 67 | 42 | 110 | 127 | 56 | 30 | 83 |
| Age ≥ 60 years | 14 | | 67 | 49 | 111 | 20 | 70 | 45 | 112 |
| Total bilirubin (μmol/L) | | | | | | | | | |
| Age < 40 years | 70 | | 12 | 4 | 28 | 102 | 9 | 4 | 30 |
| Age 40-59 years | 59 | | 10 | 3 | 27 | 127 | 8 | 4 | 20 |
| Age ≥ 60 years | 14 | | 9 | 4 | 42 | 20 | 8 | 5 | 16 |
